# Process intensification for O_2_‐dependent enzymatic transformations in continuous single‐phase pressurized flow

**DOI:** 10.1002/bit.26886

**Published:** 2019-01-08

**Authors:** Juan M. Bolivar, Alexander Mannsberger, Malene S. Thomsen, Günter Tekautz, Bernd Nidetzky

**Affiliations:** ^1^ Austrian Centre of Industrial Biotechnology (ACIB) Graz Austria; ^2^ Institute of Biotechnology and Biochemical Engineering, Graz University of Technology, NAWI Graz Graz Austria; ^3^ Microinnova Engineering GmbH Allerheiligen bei Wildon Austria

**Keywords:** flow microreactor, homogeneous liquid phase oxidation, oxygen‐dependent transformation, pressurized reactor, reaction intensification

## Abstract

Oxidative O_2_‐dependent biotransformations are promising for chemical synthesis, but their development to an efficiency required in fine chemical manufacturing has proven difficult. General problem for process engineering of these systems is that thermodynamic and kinetic limitations on supplying O_2_ to the enzymatic reaction combine to create a complex bottleneck on conversion efficiency. We show here that continuous‐flow microreactor technology offers a comprehensive solution. It does so by expanding the process window to the medium pressure range (here, ≤34 bar) and thus enables biotransformations to be conducted in a single liquid phase at boosted concentrations of the dissolved O_2_ (here, up to 43 mM). We take reactions of glucose oxidase and d‐amino acid oxidase as exemplary cases to demonstrate that the pressurized microreactor presents a powerful engineering tool uniquely apt to overcome restrictions inherent to the individual O_2_‐dependent transformation considered. Using soluble enzymes in liquid flow, we show reaction rate enhancement (up to six‐fold) due to the effect of elevated O_2_ concentrations on the oxidase kinetics. When additional catalase was used to recycle dissolved O_2_ from the H_2_O_2_ released in the oxidase reaction, product formation was doubled compared to the O_2_ supplied, in the absence of transfer from a gas phase. A packed‐bed reactor containing oxidase and catalase coimmobilized on porous beads was implemented to demonstrate catalyst recyclability and operational stability during continuous high‐pressure conversion. Product concentrations of up to 80 mM were obtained at low residence times (1–4 min). Up to 360 reactor cycles were performed at constant product release and near‐theoretical utilization of the O_2_ supplied. Therefore, we show that the pressurized microreactor is practical embodiment of a general reaction‐engineering concept for process intensification in enzymatic conversions requiring O_2_ as the cosubstrate.

**List of symbols**
*E*: amount (concentration) of enzyme catalyst, mg/ml
*E*_imm_: amount of immobilized activity, calculated from the immobilization balance U/g
*E*_obs_: amount of immobilized activity, obtained from direct initial‐rate measurement with the insoluble enzyme preparation, U/g
*F*_tot_: total flow rate, ml/min
*k*_L_*a*: volumetric mass transfer coefficient, min^−1^
[O*_2_]: O_2_ equilibrium concentration in solution, mM
OTR: O_2_ transfer from the gas to the liquid phase, mM/min
*STY*: space–time yield, mM/min
*V*: enzymatic reaction rate, μmol·ml^−1^·min^−1^
*V*_ref_: enzymatic reaction rate obtained with an amount of enzyme *E* under reference conditions (25°C, air‐saturated solution atmospheric pressure), μmol·ml^−1^·min^−1^
*F*_G_: gas flow rate, ml/min
*F*_L_: liquid flow rate, ml/min
TOF: turnover number/hr, mole product/mole catalyst/hr
TON: turnover number, mole product/mole catalyst used
[P]: product concentration, mM
τ_res_: residence time, min

## 1 INTRODUCTION

Advanced process technologies for chemical production are increasingly built on process intensification and continuous processing as the central pillars of their development (Adamo et al., 2016; Clomburg, Crumbley, & Gonzalez, 2017; Hessel, Kralisch, Kockmann, Noël, & Wang, 2013; Wiles & Watts, 2014). In this context, biocatalysis is promising to enable cleaner, safer and more energy‐efficient process technologies (Sheldon & Pereira, 2017; Sheldon & Woodley, 2018). Oxidative transformations represent an area of the chemical production in which biocatalysis is expected to have a profound impact (Dong et al., 2018; Gemoets et al., 2016). A strong oxidant (e.g., O_2_) is often required in these transformations, so running them safely and with high chemical selectivity is a difficult problem (Gemoets et al., 2016; Hone, Roberge, & Kappe, 2017). Prowess to combine reactivity with selectivity in O_2_‐dependent conversions performed under mild reaction conditions makes enzymes interesting candidates for use as oxidation catalysts in process chemistry applications (Dong et al., 2018; Martínez et al., 2017; Romero, Gómez Castellanos, Gadda, Fraaije, & Mattevi, 2018). However, the biocatalysis happens in water and supplying O_2_ to an aqueous environment faces several well‐known restrictions. In fact, the main parameters of reaction efficiency (product concentration, space–time yield (*STY*), and catalyst turnover) all depend on, and are often severely limited by, how effectively O_2_ is made available within the liquid phase (Gemoets et al., 2016; Pedersen, Rehn, & Woodley, 2015). In addition, it is paramount that the reactor design and the preparation of the enzyme used (e.g., immobilized enzyme and whole cell) both are brought in good accordance with the requirements of O_2_ supply to the continuous biotransformation envisaged (Dong et al., 2018; Gemoets, Hessel, & Noël, 2016).

The rate of O_2_ transfer from the gas to the liquid phase (OTR) is conveniently analyzed with Equation (1), where *k*_L_ *a* (min^−1^) is the volumetric mass transfer coefficient, and ([O*_2_]−[O_2_]) is deviation from the equilibrium concentration ([O*_2_]) that is determined by Henry's law,

(1) $$\text{OTR}=\frac{d[O_{2}]}{\mathrm{dt}}=k_{L}a\left(\left[O_{2}^{*}\right]-\left[O_{2}\right]\right).$$

The air‐saturated [O*_2_] at atmospheric pressure and 25°C is only 0.25 mM which poses a clear limitation from thermodynamics upon the attainable OTR under these conditions. Enzyme *K*_m_ values for O_2_ fall broadly above the actual [O_2_] at steady state (Pollegioni and Molla, 2011; Romero et al., 2018). Therefore, this implies an enzymatic reaction rate strongly dependent upon [O_2_]. The two main process variables, OTR and reaction rate, thus show complex interdependence. Immediate consequence is that a trade‐off exists between optimum utilization of the enzyme activity at high [O_2_] and maximum OTR at [O_2_] ∼0. In practice, therefore, *STYs* of O_2_‐dependent enzymatic transformations were usually low, typically below 10 mM/hr (Karande, Schmid, & Buehler, 2016; Toftgaard Pedersen et al., 2015; Tomaszewski, Schmid, & Buehler, 2014). These *STY*s are significantly smaller than the maximum OTR of 100–200 mM/h obtainable in conventional reactors for gas–liquid contacting (Pedersen et al., 2015; Toftgaard Pedersen et al., 2015). The reactors used previously showed *k*_L_ *a* values typically in the range of 1–10 min^−1^ (Garcia‐Ochoa & Gomez, 2009; Lapkin & Plucinski, 2009).

Various engineering strategies have been devised for process intensification in O_2_‐dependent biocatalysis (Gemoets et al., 2016; Karande et al., 2016; Mallia & Baxendale, 2016; Utikar & Ranade, 2017). The *k*_L_ *a* was common target in an overall approach aimed at OTR optimization. Microreaction technology offers different ways of gas–liquid contacting with high efficiency (Karande et al., 2016; Kashid, Renken, & Kiwi‐Minsker, 2011; Stone, Hilliard, He, & Wang, 2017; Utikar & Ranade, 2017; Yue, 2018). The specific surface area (*a*), the mass transfer coefficient (*k*_L_) or both are enhanced in consequence of the reactor's internal microstructure and the resulting fluidics of the two‐phase flow (Brzozowski, O'Brien, Ley, & Polyzos, 2015; Dencic, Hessel, et al., 2012; Dencic, Meuldijk, et al., 2012; Utikar & Ranade, 2017). *k*_L_ *a* values of up to 30 min^−1^ were reported for segmented gas–liquid flow in microchannels (Kashid et al., 2011). A falling‐film microreactor operated in continuous countercurrent gas–liquid flow showed a *k*_L_ *a* of 450 min^−1^ (Bolivar, Krämer, Ungerböck, Mayr, & Nidetzky, 2016). These developments notwithstanding, important engineering problems remain. Besides showing good OTR characteristics, biocatalytic microreactors must enable effective use of the enzyme. The enzyme should be stable and highly active. It should be suitable for recycling under conditions of the continuous biotransformation. To reach high substrate conversion at good *STY* and high enzyme turnover, the reactor must enable a residence time that is matched to the characteristic time of the biotransformation considered (Chapman, Cosgrove, Turner, Kapur, & Blacker, 2018; Jones, McClean, Housden, Gasparini, & Archer, 2012; Karande et al., 2016; Ringborg, Toftgaard Pedersen, & Woodley, 2017; Toftgaard Pedersen et al., 2017; Tomaszewski, Schmid, et al., 2014; Tomaszewski, Lloyd, Warr, Buehler, & Schmid, 2014; van Schie et al., 2018). Only to mention, complex interdependence of the residence time, the mass transport (dependent on mass flows) and the product formation (dependent on gas hold‐up) renders reaction optimization in segmented gas–liquid flow a rather difficult task. Finally, none of the microreactors considered previously (Karande et al., 2016; Tamborini, Fernandes, Paradisi, & Molinari, 2018) was successful in disentangling the enzymatic reaction rate from the OTR. Requirement for optimum use of the enzyme activity is a steady‐state concentration of O_2_ ([O_2_]_opt_) surpassing the enzyme *K*_m_ by roughly one magnitude order. Therefore, this implies the clear need for new process windows to be exploited for biocatalytic oxidations by O_2_.

Any robust process technology for boosting the [O_2_] available in bulk solution for the enzymatic reaction will have to go via Henry's law, hence the partial pressure of O_2_ in the gas phase. A pressurized reactor system is therefore required, irrespective of whether the soluble O_2_ is transferred from a gas phase (Gemoets, Hessel, et al., 2016; Lapkin & Plucinski, 2009) or is generated within the liquid phase from dissolved H_2_O_2_ (Chapman et al., 2018). Although not often used in biocatalysis, it is customary in industrial chemistry to run gas–liquid conversions under elevated pressure (Keybl & Jensen, 2011). Here, therefore, we developed an instrumented pressurized flow reactor for continuous enzymatic transformations in a single liquid phase at substantially enhanced O_2_ concentration. At 34 bar of pressure, the available [O_2_] was intensified 170‐fold (43 mM). With 80 mM of the product formed under these conditions, the need for gas–liquid O_2_ transfer during the biotransformation was eliminated. Spatiotemporal decoupling of the O_2_ transfer from the enzymatic conversion represents a new engineering paradigm for O_2_‐dependent biocatalysis. We use reactions of glucose oxidase (GOX) and d‐amino acid oxidase (DAAO) to demonstrate that kinetic limitations (e.g., high *K*_m_ for O_2_ of DAAO) are overcome effectively by moving to the high‐pressure/high [O_2_] range. We show that for each enzyme there exists a process window (not accessible to ambient pressure reactors) in which the specific oxidase activity and the *STY* can be enhanced simultaneously. A packed‐bed format of the pressurized flow reactor was established to make possible a continuous transformation by immobilized enzymes. With coimmobilized enzymes (oxidase and catalase) working stably in the absence of a gas–liquid interface, the reactions could be performed at a constant conversion for up to 360 reactor cycles. We thus demonstrate that the pressurized flow reactor is a powerful engineering tool for process intensification in O_2_‐dependent biochemical conversions.

## 2 MATERIALS AND METHODS

### 2.1 Materials

Porous polymethacrylate particles were used as enzyme carriers. They were gifts of Resindion (Milano, Italy). Sepabeads EC‐EP/M (10–20 nm pore diameter; 200–500 μm particle diameter; 55% water content) and ReliZyme EP403/M (40–60 nm pore diameter; 200–500 μm particle diameter, 55% water content) harbor epoxy surface groups. Reaction with polyethylenimine (PEI; see Supporting Information for details) introduced surface amino groups. The resulting carriers are referred to as Sep‐PEI and Rel‐PEI. ReliSorb 405/EB particles (80–100 nm pore diameter; 200–500 μm particle diameter; 65% water content), in short Rel‐sulfonate, harbor surface alkyl‐sulfonate groups. Throughout the paper, carrier mass always refers to the wet particles. Chemicals and reagents were of analytical grade from Roth (Karlsruhe, DE, Germany) or Sigma‐Aldrich (Vienna, Austria).

### 2.2 Enzyme preparations used

#### 2.2.1 Enzymes

The grade II GOX (β‐d‐glucose:oxygen 1‐oxidoreductase; EC 1.1.3.4) from *Aspergillus niger* was from Sigma‐Aldrich. The DAAO from *Trigonopsis variabilis* (d‐amino‐acid:oxygen oxidoreductase; EC 1.4.3.3) was used (Bolivar & Nidetzky, 2012). A chimeric form of the enzyme was used that had the binding module *Z*_basic2_ fused to its N‐terminus (Wiesbauer, Bolivar, Mueller, Schiller, & Nidetzky, 2011). Production of DAAO was done as described in earlier work (Wiesbauer et al., 2011). Catalase (CAT; hydrogen‐peroxide:hydrogen‐peroxide oxidoreductase; EC 1.11.1.6) was used from different sources. One was the commercial enzyme from bovine liver (*Bl*CAT; Sigma‐Aldrich), the other was from *Bordetella pertussis* (*Bp*CAT) and was obtained as a N‐terminal fusion protein with *Z*_basic2_ (Bolivar, Schelch, Pfeiffer, & Nidetzky, 2016). Note that *Bl*CAT and *Bp*CAT were chosen in consideration of the planned coimmobilization of oxidase and CAT. *Bl*CAT can be conveniently coimmobilized with GOX (Hernandez, Berenguer‐Murcia, Rodrigues, & Fernandez‐Lafuente, 2012). Due to the presence of *Z*_basic2_ in both enzymes, the coimmobilization of DAAO and CAT was done effectively using *Bp*CAT.

#### 2.2.2 Assays

Activities of free and immobilized enzymes were determined from initial rate measurements (30°C; 50 mM air‐saturated potassium phosphate buffer; Bolivar, Consolati, Mayr, & Nidetzky, 2013; Bolivar, Schelch, Mayr, & Nidetzky, 2014). One unit of enzyme activity is the enzyme amount consuming 1 µmol O_2_/min (GOX, pH 7.0, 100 mM glucose; DAAO, pH 8.0, 100 mM d‐Met) or 1 μmol H_2_O_2_/min (CAT, pH 7.0, 10 mM H_2_O_2_). The specific activities of the enzymes used were: GOX, 125 U/mg; DAAO (purified), 71 U/mg; *Bl*CAT, 5,000 U/mg; *Bp*CAT (purified), 60,000 U/mg. The commercial GOX and *Bl*CAT preparations were used without further purification. Unless mentioned, DAAO and *Bp*CAT were used as *Escherichia coli* cell extract containing the recombinantly expressed enzyme (DAAO, 12 mg protein/ml, 26 U/ml; *Bp*CAT, 21 mg protein/ml, 5,300 U/mg).

#### 2.2.3 Immobilization

GOX and *Bl*CAT were coimmobilized on Sep‐PEI and Rel‐PEI based on ionic adsorption of the enzymes. A reported protocol was used with slight modifications described in the Supporting Information Methods S1). DAAO and *Bp*CAT were coimmobilized on Rel‐sulfonate. Previously reported procedure (Bolivar, Schelch, et al., 2016) was used. The DAAO was immobilized before the *Bp*CAT. Of note, the enzyme immobilization involved affinity‐like ionic adsorption via the Z_basic2_ module. This confers high selectivity to the enzyme immobilization directly from the cell extract and also ensures enzyme‐surface interaction in a defined molecular orientation via Z_basic2_ (Bolivar, Schelch, et al., 2016; Wiesbauer et al., 2011). The immobilization was monitored by enzyme activity measurement, both in solution and directly on the carrier. The total activity immobilized, *E*_imm_ (U/g_carrier), was calculated from the activity balance in solution. *E*_obs_ (U/g_carrier) is the directly measured activity of the enzyme immobilized on the carrier. An *E*_obs_ lower than *E*_imm_ is explainable by effect of the immobilization on the intrinsic enzyme activity, diffusional effects or both.

### 2.3 Pressurized flow reactor design and set‐up

**Figure 1:**
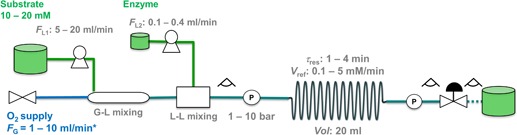
The flowchart of the high‐pressure reactor operated with soluble enzymes is shown. The system comprised the reactor coil, a mass‐flow controller for gas delivery, two pumps controlling liquid inflow, two flow‐through pressure sensors at the inlet and the outlet of the reactor unit, and a backpressure regulator. The reactor components were made of stainless steel. Observation windows made from Teflon tubes were included as indicated [Color figure can be viewed at wileyonlinelibrary.com]

The reactor in Figure 1 was developed. It was constructed by Microinnova Engineering GmbH (Allerheiligen bei Wildon, AT). The reactor used for studies of the soluble enzymes comprised a stainless‐steel reaction coil (length, 5.4 m; nominal diameter, 0.3175 cm; volume, 19.97 ml). The packed‐bed reactor used with immobilized enzymes comprised a Supelco column (ID × OD × L: 10 mm × 12 mm × 25 cm) filled with 10 g of carrier particles. The final volume of the packed bed was 14 ml. The total bed porosity (ε) was calculated from the inter and intraparticle porosities as ε = 0.58 The liquid flow was delivered from one or two analytical isocratic pumps (model Azura P2.1S; Knauer Wissenschaftliche Geräte GmbH, Berlin, DE) equipped with 10 or 50 ml pump heads. When soluble enzymes were used, substrate and enzyme solutions were delivered from two separate lines and brought together using a model CPMM‐R600/12 Caterpillar Micromixer (profile width, 600 μm) from IMM (Mainz, DE). Pure O_2_ gas (99.9%) was supplied using a Bronkhorst EL‐flow mass‐flow controller (Bronkhorst High‐Tech B.V., AK Ruurlo, The Netherlands). Note that the O_2_ mass flow is referred to as volumetric gas flow under standard conditions (1 bar, 25°C). As shown in Supporting Information Figure S1, standard volumetric flows are equivalent to mass flows or real volumetric flows under the operating conditions used. A mixing column (length, 30 cm; nominal diameter 1/4″) packed with glass particles (diameter, 3.0 mm) was used to merge the gas and liquid flows into a single liquid phase under pressurized conditions. For recording the operating pressure, two piezo‐resistive pressure transmitters (model, PA 21G″; pressure range, 0–60 bar; Keller AG, Winterthur, CH, Switzerland) were used. A Bronkhorst EL‐PRESS series backpressure controller was used to adjust the system pressure. Pressure is reported in absolute values. Interactions between components of the reactor system were managed by custom computer software, developed at Microinnova Engineering GmbH based on LabView (National Instruments, Austin, TX).

### 2.4 Pressurized flow reactor operation

All reactions were performed at 24 ± 1°C using 50 mM potassium phosphate buffer (GOX, pH 7.0; DAAO, pH 8.0). The system pressure (atm‐34 bar) and the flow rates of liquid (*F*_L_ = 1–20 ml/min) and gas (*F*_G_ = 1–25 ml/min) were varied. Substrate concentrations were also varied (10 − 100 mM). Unless mentioned, glucose was used for GOX, d‐Met for DAAO. Samples were collected at the reactor outflow, diluted in hydrochloric acid to inactivate any enzyme present, and analyzed by HPLC (Supporting Information Methods S2). In the case of the DAAO reaction, besides analysis of d‐Met and its oxidative deamination (α‐keto‐acid) product, the samples were also analyzed for potential decarboxylation products. Glucose consumption was additionally measured using a commercial test kit (Glucose Hexokinase UV; DIPROmed, Weigelsdorf, Austria; Schwarz, Thomsen, & Nidetzky, 2009). The α‐keto‐acid product was additionally measured by a colorimetric assay (Bolivar, Schelch, Mayr, & Nidetzky, 2015).

The reactor operation started with liquid flow at atmospheric pressure. Samples were collected at steady state to confirm that product formation (∼0.25 mM) was as expected from the low [O_2_*] present under these conditions. It was confirmed that the liquid flow at the reactor outlet was completely deoxygenated due to the enzymatic reaction. The pressure was then adjusted using the backpressure regulator and product formation was monitored over time until steady‐state flow was reached. Finally, the O_2_ gas flow was implemented in exact correspondence to the liquid flow and the pressure applied, thus making sure according to Supporting Information Figure S1 that only a single liquid phase was present in which the O_2_ was dissolved completely. Visual inspection was used to confirm the absence of gas bubbles in the final liquid flow. The reactor was then operated at constant conditions to reach a new steady state. Enzyme stability was analyzed in control experiments under pressurized flow conditions in the absence of substrate. Samples were collected from the outflow and enzyme activities were recorded offline. At the end of the reaction, the pressure was released and the reactor operated under segmented gas–liquid flow (slug flow). When soluble enzymes were used, the substrate flow rate was in the range of 4.9–19.6 ml/min, whereas the enzyme flow rate was in the range 0.1–0.4 ml/min. The final enzyme concentration in the liquid phase varied between 0.001 and 0.14 mg/ml, corresponding to 0.1 and 18 U/ml. The substrate flow was previously mixed with O_2_ gas flow to give a single‐phase liquid flow, which was only then mixed with the enzyme flow. The total flow rate *F*_tot_ varied in the range of 2.5–20 ml/min. The concentration of product ([P]) released at steady state was measured. The *STY* was calculated as: *STY* = *F*_tot_ [P]/Vol, where Vol is the reactor volume. The enzymatic reaction rate *V* thus equals *STY*. Note that *STY* equals the initial *V* at relatively low substrate conversion.

### 2.5 Reaction kinetic analysis

The reaction kinetics of the soluble oxidases were described using Equation (2) which is the rate equation for a Ping‐Pong two‐substrate enzyme mechanism. Equation (2) is known to apply to the kinetics of GOX and DAAO.

(2) $$V=\frac{d[P]}{dt}=\frac{EAct_{max}[O_{2}][S]}{\left[O_{2}][S]+K_{S}[O_{2}]+K_{O_{2}}[S\right]}.$$

In Equation (2), *V* is the reaction rate (μmol·ml^−1^·min^−1^), *S* and O_2_ are concentrations, and *K*_S_ and $K_{O_{2}}$ are the corresponding Michaelis constants. Act_max_ is the enzyme specific activity at saturation with both substrate and O_2_. *V*_max_ is the maximum reaction rate obtainable with a certain amount of catalyst *E* (mg/ml). *V*_ref_ is the initial reaction rate obtained with the same *E* under reference conditions (25°C; air‐saturated solution at atmospheric pressure; 50 mM substrate). The ratio *V*/*V*_ref_ accordingly is the degree of *kinetic intensification* of the reaction achieved under pressurized flow conditions. Note that *V*_ref_ is a rigorously defined and thus unambiguously applicable (reference) parameter to assess the kinetic intensification due to reaction at elevated pressure.

Equation (3) describes the O_2_ consumption in a batch biotransformation that involves continuous O_2_ transfer from the gas phase. The differential equation solver Berkeley Madonna (Version 8.3.18) was used for modeling and simulation.

(3) $$\text{OTR}\;=\;\frac{d[O_{2}]}{\mathrm{dt}}=k_{L}a\left(\left[O_{2}^{*}\right]-\left[O_{2}\right]\right).$$

## 3 RESULTS AND DISCUSSION

### 3.1 Pressurized flow reactor operated with soluble enzymes

The operating pressure of the single‐phase reactor was initially set to 10 bar. The resulting [O_2_] in solution (∼12 mM) was saturating for both GOX and DAAO. Kinetic intensification of the enzymatic reactions, compared to the atmospheric pressure reference ([O_2_] = 0.25 mM), was thus maximized under these conditions (for discussion, see Section 3.2). Therefore, *F*_L_ and *F*_G_ were adjusted to form a single liquid phase (Supporting Information Figure S1) whose flow rate corresponded to an average residence time (τ_res_) of 1 min. The Supporting Information Methods S3 summarizes the hydrodynamic characterization of the liquid flow in terms of dimensionless parameters. The flow was laminar based on low Reynolds number (≤226). It furthermore featured low axial dispersion due to the small diameter of the coiled tube used (for details, see Supporting Information). Within the range of τ_res_ used in later experiments (1–4 min), therefore, the assumption of plug flow was justified. Operating the flow reactor in the experiments described below involved a lower‐limit *STY* of ∼1 mM/min. This *STY* was chosen as reference point for an analysis of reaction intensification based on literature (Chapman et al., 2018; Jones et al., 2012; Karande et al., 2016; Toftgaard Pedersen et al., 2017; Tomaszewski, Schmid, et al., 2014; Tomaszewski, Lloyd, et al., 2014; van Schie et al., 2018), as further discussed later.

Initial experiments in the single‐phase pressurized flow reactor performed with GOX (pressure range, 2–10 bar; τ_res_, 1 min) revealed a steady‐state product concentration of only ∼1 mM. The product concentration formed was much lower than the concentrations of substrate (20 mM) and O_2_ available (Supporting Information Figure S2). Increase of τ_res_ to 2 or 4 min hardly affected the concentration of the product released (Supporting Information Figure S2). We considered that the GOX might become inactivated by the H_2_O_2_ formed in the reaction. The use of catalase was therefore implemented (Hernandez et al., 2012). All experiments were performed employing *Bl*CAT in a U amount exceeding that of the oxidase used (GOX, DAAO) by about 10^3^‐fold. Note that the activity ratio of oxidase and catalase was not optimized for an economic use of the catalase. Efficient and complete removal of the H_2_O_2_ was the goal to be achieved here.

**Figure 2:**
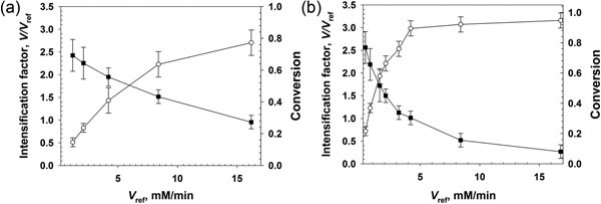
Conversion of glucose catalyzed by soluble GOX in the pressurized flow reactor at different enzyme concentrations is shown. The substrate conversion (open circles) and the kinetic intensification factor *V/V*_ref_ (closed squares) are depicted. The enzyme concentration used is expressed as *V*_ref_ (Equation (2)) at 20 mM glucose. The pressure was 10 bar. (a) τ_res_ = 1 min. (b) τ_res_ = 4 min. All experiments used *Bl*CAT in a U amount exceeding that of GOX by about 10^3^‐fold. The data are mean (*SD*) values from multiple experiments (*N* ≥ 5) performed at steady state

In Figure 2 we show experiments performed at 10 bar pressure using different GOX concentrations. Variation in the enzyme concentration is expressed as change in the maximum reference rate *V*_ref_ (0.2 − 16.2 mM/min). The kinetic intensification factor (*V*/*V*_ref_) was high (∼2.5) at the lowest *V*_ref_ and decreased to ∼1 (τ_res_ = 1 min; panel A) or below unity (τ_res_ = 4 min; panel B) at high *V*_ref_. Note: a *V* equal to or smaller than *V*_ref_ is possible when the *V* was determined at high substrate conversion. *V*_ref_ by contrast is an *initial* rate. The glucose consumption increased with increasing *V*_ref_, allowing for a near‐complete conversion of the 20 mM substrate present when τ_res_ was 4 min (Figure 2b), the dissolved concentration of O_2_ was 12 mM. As expected from the presence of CAT, the H_2_O_2_ was fully degraded. Partial recycling of O_2_ from H_2_O_2_ improved the efficiency and economy of O_2_ utilization in the enzymatic reaction. The overall reaction becomes, substrate + 0.5 O_2_ → oxidized product, under these conditions. We analyzed the dependence of *V*/*V*_ref_ on the applied pressure in the range of 3–10 bar. As shown in Supporting Information Figure S3, the dependence appeared hyperbolic, with most of the increase in *V*/*V*_ref_ happening at pressures below 5 bar.

**Figure 3:**
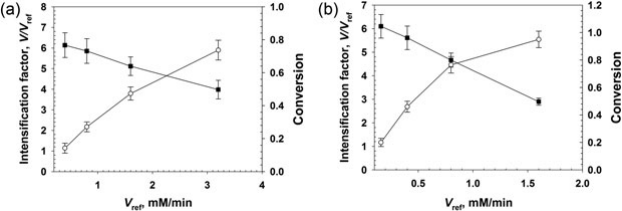
Conversion of d‐Met catalyzed by soluble DAAO in the pressurized flow reactor at different enzyme concentrations. Conversion (open circles) and kinetic intensification factor *V/V*_ref_ (closed squares) are shown. The enzyme concentration used is expressed as *V*_ref_ (Equation 2) at 20 mM d‐Met. Pressure was 10 bar. (a) τ_res_ = 1 min. (b) τ_res_ = 4 min. All experiments used *Bp*CAT in a U amount exceeding that of DAAO by about 10^3^‐fold. The data are mean (*SD*) values from multiple experiments (*N* ≥ 5) performed at steady state. CAT: catalase; DAAO: d‐amino acid oxidase

Study of the DAAO reaction at high‐pressure flow conditions is summarized in Figure 3. As with GOX, the *V*_ref_ was varied to allow for full substrate conversion (20 mM d‐Met) at the high τ_res_ used (4 min). The maximum *V*/*V*_ref_ at low conversion was ∼6. Contrary to the GOX reaction, the *V*/*V*_ref_ remained substantially above unity (∼3) up to full conversion of the substrate. The *V* showed a remarkably high value between 6 and 20 mM/min.

### 3.2 Enzyme kinetic properties and reaction intensification at high pressure

**Figure 4:**
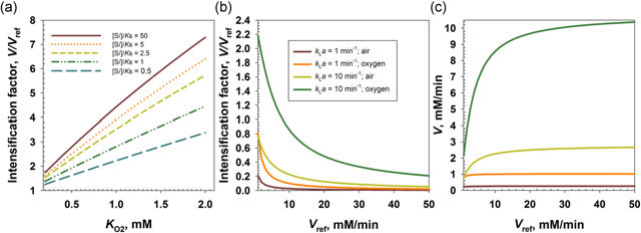
Analysis of reaction intensification for an enzyme‐catalyzed O_2_‐dependent reaction is shown. *V* and *V*_ref_ were calculated according to Equation (2). (a) Effects of intrinsic enzyme kinetics. *V* was calculated for a O_2_ concentration of 10 mM. Different [S]/*K*_S_ ratios were used in the calculation, as shown in the graph. (b, c) Effects of the enzyme concentration and of the OTR in aerated or O_2_‐gasified conditions at atmospheric pressure. Calculations were performed using Equations (2) and (3). [S]/*K*_S_ = 50; $K_{O_{2}}$ = 1 mM. The figure shows two cases of medium and high *k*_L_ *a*. OTR: O_2_ transfer from the gas to the liquid phase [Color figure can be viewed at wileyonlinelibrary.com]

Differences in enzyme kinetic properties explain the greater benefit from high‐pressure reaction conditions on the DAAO reaction as compared to the GOX reaction. The DAAO has a relatively higher *K*_m_ for O_2_ (∼1.2 mM; Pollegioni, Buto, Tischer, Ghisla, & Pilone, 1993; Rosini, Molla, Ghisla, & Pollegioni, 2011) than GOX (∼0.3–0.5 mM; Gibson, Swoboda, & Massey, 1964; Nakamura, Hayashi, & Koga, 1976). However, the substrate *K*_m_ is much lower in DAAO (d‐Met, ∼10 µM; Kubicek‐Pranz & Röhr, 1985) than it is in GOX (glucose, ∼25–75 mM; Gibson et al., 1964; Nakamura et al., 1976). We show a general analysis in Figure 4a. This reveals that the *V*/*V*_ref_ achievable at 10 bar pressure depends upon the O_2_ *K*_m_, as expected, but it also depends strongly on the ratio between the substrate concentration used and the substrate *K*_m_. Under conditions in which the enzyme is not saturated with substrate ([S]/*K*_m_ ≤2.5; cf., reaction of the GOX), the intensification effect of elevated [O_2_] is mitigated strongly (Figure 4a).

To compare reaction at high pressure with reaction under aerated or O_2_ gasified conditions at atmospheric pressure, the effect of OTR on *V*/*V*_ref_ was simulated in dependence upon the *V*_ref_. We show in Figure 4b that even at a high *k*_L_ *a* (10 min^−1^) and using pure O_2_ gas, *V*/*V*_ref_ is limited to values of ∼2.2 or smaller. The *V*/*V*_ref_ drops rapidly as the enzyme loading (*V*_ref_) is increased, implying a low degree of utilization of the enzyme activity present. OTR limits the overall productivity (*V*) to a value of 10 mM/min, as shown in Figure 4c.

### 3.3 Reactor comparison for O_**2**_‐dependent conversions

To enable comparison with reactors and reaction conditions from the literature, we summarize in Table 1 important parameters of reaction efficiency for the pressurized flow reactor. It can be shown that the pressurized reactor stands out in reaching, at the same time, a high enzyme turnover (TON, 10^4^ mole product/mole enzyme), a high *STY* (≥100 g/[L·hr]; 12 mM/min) and a high conversion (0.8–1.0). This is uniquely possible for the pressurized flow reactor because, unlike alternative reactors previously considered, it does not involve trade‐off between the [O_2_] at steady state, the gas–liquid transport rate, and the τ_res_. Besides the enhanced supply of dissolved O_2_, the pressurized reactor also involves kinetic intensification due to the increased [O_2_] (see discussion in Section 3.2 and the references given there for the enzyme *K*_m_ values). Using a segmented flow tubular reactor for enzymatic hydroxylation of *trans*‐hex‐2‐enol, van Schie et al. (2018) obtained a large TON (3 × 10^5^) at low conversion (0.1; τ_res_, ≤5 min). At a high conversion of 0.9, however, the *STY* was low (0.25 mM/min). Using a falling‐film microreactor for the GOX reaction, Illner, Hofmann, Löb, and Kragl (2014) obtained *STY* of ~80 mM/min at 20–30% of conversion at low TON (2 × 10^3^). Using the same type of reactor, we reported a *STY* of up to 45 mM/min, however, at low TON of soluble GOX and low conversion (Bolivar, Krämer, et al., 2016). In both cases (Bolivar, Krämer, et al., 2016; Illner et al., 2014), the equivalent TOF (TON/residence time) was below the TOF of the catalyst at air‐saturated conditions (~1 × 10^4^ min^−1^). Boundaries in terms of *STY* and catalyst productivity were theoretically discussed in a seminal study by Dencic and coworkers (Dencic, Meuldijk, et al., 2012). Using a tube‐in‐tube reactor for hydroxylation of 2‐hydroxybiphenyl, Tomaszewski and coworkers (Tomaszewski, Schmid, et al., 2014 and Tomaszewski, Lloyd, et al., 2014) obtained a TON of 6 × 10^3^ and a *STY* of 1.58 mM/min. These findings from literature exemplify the fundamental problem (Figure 4, panels b and c) that in reactors requiring gas–liquid transfer there is trade‐off between OTR and *V*, hence *STY*. Chapman et al. (2018) suggested that one might eliminate the requirement for gas–liquid transport through the in situ generation of O_2_ from H_2_O_2_. A number of studies before Chapman et al. (2018) have elaborated on the concept (Bolivar, Schelch, et al., 2016; Schneider, Dorscheid, Witte, Giffhorn, & Heinzle, 2012; Van Hecke et al., 2009; Yoshimoto & Higa, 2014). To overcome the limit of OTR at *ambient pressure* (for use at high pressure, see Figures 2 and 3), the H_2_O_2_‐based oxygenation would have to be performed strictly under conditions of rate‐limiting formation of O_2_ (Bolivar, Schelch, et al., 2016). Besides issues of enzyme stability caused by a significant level of H_2_O_2_ present at steady state under such conditions, keeping the balance between the enzyme activities present as to prevent O_2_ gas formation seems challenging. Anyway, in the study of Chapman et al. (2018), the maximum *STY* was 8 mM/min, which is below the standard OTR limit using gasification with pure O_2_ (Figure 4c), the TON was ~2 × 10^3^.

**Table 1:** Summary of the performance metrics of the single‐phase pressurized reactor operated with free enzymes

	TON^a^ (10^4^ mole product/mole enzyme)		Catalyst productivity^b^ (g product/g enzyme)		*STY* (g/[L·hr])	
	*X* = 0.1	*X* = 0.9	*X* = 0.1	*X* = 0.9	*X* = 0.1	*X* = 0.9
GOX	2.8	1.1	65	23	37	182
DAAO	2.5	1.0	76	48	22	113

*Note*. DAAO: d‐amino acid oxidase; GOX: glucose oxidase; *STY*: space–time yield; TON: turnover number; *X*, conversion.

^a^ TON is the ratio of the product concentration and the molar enzyme concentration used. The molar enzyme concentration was calculated from the *E* and the molecular mass of the monomer (GOX, 80.0 kDa; DAAO, 46.3 kDa). Results are shown for a τ_res_ of 1 min.

^b^ The catalyst productivity was calculated from the mass concentration of product and the amount of enzyme used *E*. Results are shown for a τ_res_ of 1 min.

### 3.4 Implementation of pressurized packed‐bed reactor

**Figure 5:**
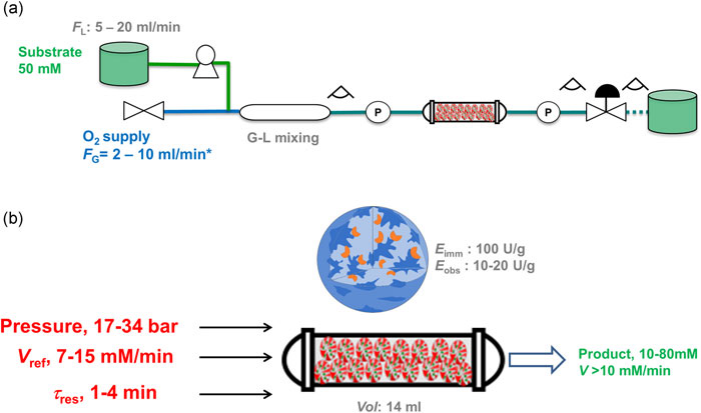
The flowchart of the high‐pressure reactor operated with immobilized enzymes is shown. (a) Packed‐bed reactor integrated into the pressurized reactor plant. (b) Design of the reactor operation and the relevant reactor performance parameters. Green: target values; Red: design variables chosen to satisfy the targets; Grey: reactor dimensions and properties of the catalyst used [Color figure can be viewed at wileyonlinelibrary.com]

When performing enzymatic transformations in flow, it is customary to use the enzyme in a form suitable for continuous processing with enzyme recycling (Karande et al., 2016; Tamborini et al., 2018). Enzyme immobilization on a solid support is most commonly used to that end. Despite significant advances in flow reactor applications (Karande et al., 2016; Tamborini et al., 2018), study of the intensification of O_2_‐dependent conversions using immobilized enzymes is lacking. In Figure 5 we show the pressurized flow reactor for use with immobilized enzymes. The overall reactor design reflects the idea of expanding the current boundaries of reactor performance in terms of *V* (10 mM/min; Dencic, Hessel, et al., 2012 and Dencic, Meuldijk, et al., 2012). Our choice of carrier material for enzyme immobilization took into account specifically that the pressure drop over a packed bed (volume, 14 ml; length, 13.3 cm) should be low; a sufficient amount of enzyme activity should be bound to the carrier; and the enzyme attachment on the carrier surface should be stable during continuous operation. The practical τ_res_ was in the range of 1–4 min. As shown in the Supporting Information Methods S3, hydrodynamic characterization of the liquid flow indicated laminar flow conditions (Reynolds number, 2.3–4.7) with low axial dispersion.

The *V*_ref_ range applicable to the immobilized‐enzyme flow reactor depends upon the processing objective(s) (e.g., degree of conversion, *STY*) in relation to the τ_res_ on the one hand, and upon the immobilization efficiency on the other. If we define as the processing objective the full conversion of [O_2_] in the range 10–40 mM within the set τ_res_ range, the required *V* will be between 10 and 40 mM/min. We considered how to achieve this *V* under the additional constraint that *V/V*_ref_ should be greater than unity. We show in the Supporting Information that the enzyme immobilizates obtained a maximum *E*_obs_ of 15 U/g (GOX; Supporting Information Figure S4) and 21 U/g (DAAO; Supporting Information Figure S5) when the total amount of enzyme activity loaded (*E*_imm_) was in the range of 100–200 U/g. To avoid conditions in which the *E*_obs_ lower than *E*_imm_ involved substantial rate limitation from diffusion into the solid carrier, we chose the lowest *E*_imm_ (100 U/g; ~1 mg protein/g carrier) still giving the maximum value of *E*_obs_.

**Figure 6:**
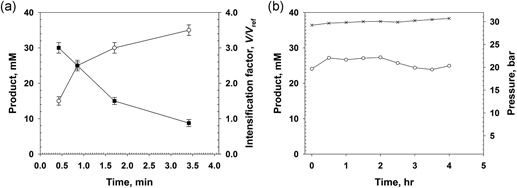
Conversion of glucose by GOX coimmobilized with *Bl*CAT in the pressurized flow reactor. The glucose concentration used was 50 mM. (a) Product concentration (open circles) and the *V/V*_ref_ ratio (closed squares) dependent upon variation of τ_res_. The data are mean (SD) values from multiple experiments (*N* ≥ 3) performed at steady state. (b) Time course of product formation (open circles) at the indicated pressure (cross symbols). The pressure drop was lower than 0.5 bar under the operation conditions. Experiments were performed at τ_res_ = 60 s. The data shown are representative of multiple time‐course experiments performed in this study. The enzyme coimmobilizate contained GOX and *Bl*CAT immobilized on Sep‐PEI; for GOX, *E*_imm_ = 100 U/g, *E*_obs_ = 10 U/g; for *Bl*CAT, *E*_imm_ = 10,000 U/g, *E*_obs_ = 400 U/g. CAT: catalase; GOX: glucose oxidase; PEI polyethylenimine

In Figure 6a, we summarize flow reactor studies at 34 bar pressure for GOX coimmobilized with *Bl*CAT. The [P] released at steady state and the *V/V*_ref_ are shown dependent upon the τ_res_. It is worth noting that no enzyme activity was eluted during continuous reactor operation over several hours. The [P] increased from 15 mM to 35 mM on increasing the τ_res_ from 0.5 to 3.5 min. The *V/V*_ref_ started out at ∼3 at low τ_res_ and decreased to ∼1 at high τ_res_. Effects of depletion of substrate and O_2_ on the enzyme kinetics explain the nonlinear dependence of [P] upon τ_res_ and the consequent lowering of the degree of reaction intensification (*V/V*_ref_).

In Figure 6b, we show that the pressurized flow reactor could be operated stably for 240 (total run time/τ_res_ = 240/1) reactor cycles. After 4 hr, the pressure was released and the flow reactor operated at the same flow conditions (i.e., *F*_L_ and *F*_G_) as in Figure 6b, but at atmospheric pressure. This resulted in a gas–liquid slug flow, and the [P] released was decreased to only about 10–15% of the [P] released under the pressurized flow conditions. The experiment at atmospheric pressure may be taken as a relevant control. However, we wish to emphasize that in this control the gas–liquid slug flow was not optimized.

**Figure 7:**
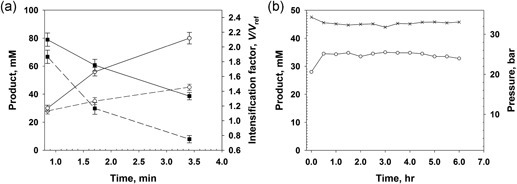
Conversion of d‐Met catalyzed by DAAO coimmobilized with *Bp*CAT in pressurized flow reactor. (a) Product concentration (open circles) and *V/V*_ref_ (closed squares) dependent upon variation of τ_res_. Data are mean (*SD*) values from multiple experiments (*N* ≥ 3) performed at steady state. Dotted and continuous lines indicate 50 and 100 mM d‐Met, respectively. (b) Time course of stable product formation (open circles) at the indicated pressure (cross symbols). The pressure drop was was lower than 0.5 bar at the operation conditions. The d‐Met concentration used was 100 mM. Experiments were performed at τ_res_ = 60 s. The data shown are representative of multiple time‐course experiments performed in this study. The coimmobilizate contained DAAO and *Bp*CAT immobilized on Rel‐sulfonate; for DAAO, *E*_imm_ = 100 U/g, *E*_obs_ = 17 U/g; for *Bp*CAT, *E*_imm_ = 20000 U/g, *E*_obs_ = 350 U/g. CAT: catalase; DAAO, d‐amino acid oxidase

In Figure 7, we show results of flow reactor studies of DAAO coimmobilized with *Bp*CAT. Product analysis by HPLC showed that the d‐Met substrate was cleanly converted to the α‐keto‐acid product. No decarboxylation product was observed under the conditions used (for details, see Supporting Information Figures S6 and S7). The dependence of [P] upon τ_res_ (Figure 7a) showed that a substantial amount of product (∼30 mM) was formed at the lowest τ_res_ of 0.75 min. However, a further increase in τ_res_ to 3.5 min did not improve the production proportionally ([P] = ∼42 mM). Furthermore, the *V/V*_ref_ was relatively low as compared to expectation from the results obtained with the soluble DAAO. Increase in the d‐Met substrate concentration from 50 to 100 mM caused enhancement of the product formation dependent upon the τ_res_. It thus enabled complete conversion of the entire O_2_ available in the system, to release about 80 mM of product. Considering reaction in a single‐liquid phase dependent on O_2_, this truly is a remarkable concentration of product formed. The result reveals a highly efficient use of the dissolved O_2_. In contrast, when the reactor was operated at the same conditions (i.e., *F*_L_ and *F*_G_) as in Figure 7b, but at atmospheric pressure, the [P] released was decreased to less than 10% of the [P] released under the pressurized conditions (data not shown). Figure 7a also shows that the increase of the substrate concentration improved the *V/V*_ref_, but it was still low in comparison to the *V/V*_ref_ obtained with the soluble DAAO. There is good evidence that the DAAO reaction with immobilized enzyme under the conditions used was strongly limited by diffusion, as follows.

Under air‐saturated conditions at atmospheric pressure, reaction of a DAAO immobilizate as used here is known to be severely restricted by O_2_ diffusion into the catalyst particle (Bolivar et al., 2014). Assuming that the increase in [O_2_] at high pressure could mitigate the diffusional restrictions, one would expect for the *E*_imm_ of 100 U/g used in the experiment that the immobilized reactor would show a *V* of ~700 mM/min. This *V* implies a reaction time of just 5 s to deplete the 50 mM substrate used. It would not be surprising if mass transport became a limiting factor under these conditions, especially at high substrate conversion. To support the notion of reactor operation in the diffusional regime, we calculated the chemical engineering parameter Thiele modulus and did so for the two extreme cases that the enzymatic reaction obeys first‐ and zero‐order kinetics with respect to the limiting substrate concentration used. The Thiele modulus is a dimensionless number that sets into relation the rates of intraparticle reaction and diffusion. The calculated value of ≥10, irrespective of the reaction order assumed (see Supporting Information Figure S8), indicated a massive limitation of the observable *V* by diffusion. Therefore, the *E*_obs_ was expected to be reduced to ≤10% of the actual *E*_imm_ under these conditions (Doran, 2013). In addition, one can calculate, that for substrate to reach the center of the carrier particle when *V* is 700 mM/min, the particle radius would have to be lower than 20 µm (Supporting Information Figure S8). This however is not a practical particle size. A more detailed study of diffusional limitations in DAAO immobilizates at high‐pressure reaction conditions was left for consideration in future research. In any event, further strategies of reaction intensification with immobilized enzymes are of high interest (Bolivar, Valikhani, & Nidetzky, 2018). They have significant potential to create synergy with the “high‐pressure flow approach” developed in this study.

Stable operation of the pressurized flow reactor for 360 reactor cycles is shown in Figure 7b. Enzyme elution was not observed under the conditions used. This is worth emphasizing because both DAAO and CAT were immobilized noncovalently and in an affinity‐like fashion via the Z_basic2_ module. Parameters of reaction efficiency for both the GOX and the DAAO conversions are summarized in Table 2. The results indicate an outstanding performance of the pressurized flow reactor.

**Table 2:** Summary of the performance metrics of single‐phase pressurized reactor with immobilized enzymes analyzed at low and high conversion (*X*)

	TOF^a^ (10^5^ mole product/[mole enzyme·hr])		Catalyst productivity^b^ (g product/[g enzyme·hr])		*STY* (g/[L·hr])	
	*X* = 0.3	*X* = 0.8	*X* = 0.3	*X* = 0.8	*X* = 0.3	*X* = 0.8
GOX	1.6	0.5	440	130	250	74
DAAO	0.59	0.39	190	120	190	130

*Note*. DAAO: d‐amino acid oxidase; GOX: glucose oxidase; *STY*: space–time yield; TOF: turnover frequency.

^a^ TOF was calculated from the the product concentration (mM), *F*_tot_ and the molar amount of enzyme used. The amount of enzyme was calculated from the *E*_imm_, the mass of carrier used, the specific activity of the free enzyme, and the molecular mass of the monomer (GOX: 80.0 kDa; DAAO: 46.3 kDa). Note that TON= TOF ×  time of reactor operation (here, 6 hr).

^b^ The catalyst productivity was calculated from the mass concentration of product, *F*_tot_ and the mass amount of enzyme used. The mass amount of enzyme used was calculated from the *E*_imm_, the mass of carrier used and the specific activity of enzyme. Results are shown for a τ_res_ of 1 min.

## 4 CONCLUSION

We show in this study that the pressurized flow reactor enables new process windows for O_2‐_dependent biotransformations to be performed at significantly improved efficiency. The pressurized reactor is a unique engineering tool that effectively decouples in space and time the gas–liquid O_2_ transfer from the O_2_‐dependent reaction in solution. This decoupling facilitates reaction control and optimization in single liquid phase flow as compared to reaction in gas–liquid two‐phase flow. It allows for reaction rate intensification due to the increased [O_2_] in solution. It protects enzymes, especially soluble ones, against the denaturing contact with gas–liquid interfaces. By working at high pressure, the flow reactor makes possible that the two main principles of O_2_ supply to liquid phase, namely the gas–liquid transport and the O_2_ release from H_2_O_2_ in solution, can be effectively combined in a practical manner. We demonstrate efficient application of the pressurized reactor to continuous conversions with immobilized enzymes at very high TON (≥10^5^), *STY* (25 mM/min), and [P] (80 mM). The pressurized reactor is unique in avoiding trade‐off between these process efficiency parameters which it is difficult to manage even in the currently most advanced reactors requiring OTR at ambient pressure (Dencic, Hassel, et al., 2012; Dencic, Meuldijk, et al., 2012; Karande et al., 2016; Kashid et al., 2011). The pressurized flow reactor appears widely applicable to O_2_‐dependent biotransformations and is flexible to accommodate the various characteristics/requirements these transformations may have.

## CONFLICTS OF INTEREST

M. S. T and G. T. are employees of Microinnova Engineering GmbH with an interest in the commercial use of microreactor technology. For the remaining authors, there are no conflicts of interest.

## Supporting information

Supporting information
