# Supplementary material for: Process intensification for O2‐dependent enzymatic transformations in continuous single‐phase pressurized flow
Source: Biotechnol Bioeng. 2019 Jan 8;116(3):503–14. doi: 10.1002/bit.26886 (PMC6590253; doi:10.1002/bit.26886)
Supplement: Supplementary file 1 — Supporting information [file BIT-116-503-s001.docx]

Supporting Information

**Process Intensification for O_2_-Dependent Enzymatic Transformations in Continuous Single-Phase Pressurized Flow**

Juan M. Bolivar^1,2^, Alexander Mannsberger^2^, Malene S. Thomsen^3^, Günter Tekautz^3^, Bernd Nidetzky^1,2*^

^1^ Austrian Centre of Industrial Biotechnology (acib), Petersgasse 14, A-8010 Graz, Austria

^2^ Institute of Biotechnology and Biochemical Engineering, Graz University of Technology, NAWI Graz, Petersgasse 12, A-8010 Graz, Austria

^3^ Microinnova Engineering GmbH, Europapark 1, A-8412 Allerheiligen bei Wildon, Austria

* Corresponding author (B. N.)

Phone: +43 316 873 8400; Fax: +43 316 873 8434; E-mail: bernd.nidetzky@tugraz.at

**Supporting methods**

**S1 Carrier preparation**

Sep-PEI and Rel-PEI were prepared from Sepabeads EC-EP/M and ReliZyme EP403/M. The used protocol was adapted from Mateo et al. (2000). Carrier particles (10 g) were suspended in 100 mL of 10% (w/v) polyethyleneimine solution prepared in distilled water at pH 10.0. The mixture was gently stirred for 16 h at room temperature. The particles were washed with 100 mM sodium acetate buffer (pH 5.0) and sodium hydrogen carbonate buffer (pH 9.0). They were additionally washed with distilled water and stored in 50 mM sodium phosphate buffer (pH 7.0).

**S2 Analytics**

The concentrations of glucose and gluconic acid were determined by HPLC using a Luna® column (5 µm, NH_2_, 100 Å; Phenomenex, Aschaffenburg, DE). The analysis was performed at 40 ºC using an aqueous mobile phase that contained 20 mM phosphoric acid (pH 2.0). The flow rate was 1.5 mL/min. The retention time of glucose and gluconic acid was 2.0 min and 1.3 min, respectively, under the conditions used. Glucose was detected by a RI detector. Gluconic acid was detected by a UV/VIS detector at 205 nm. The concentrations of D-Met and α-keto-γ-(methylthio)butyric acid were determined by HPLC using a Kinetex® column (2.6 µm, C8, 100 Å; Phenomenex). Analysis was performed at 40 ºC with an aqueous mobile phase containing 5.0% acetonitrile (v/v) and 40 mM tetrabutylammonium bromide (pH 5.9). The flow rate was 1.0 mL/min. The retention time of D-Met and the α-keto-acid was 0.6 min and 3.6 min, respectively, under the conditions used. Both compounds were detected by a UV/VIS detector at 210 nm. The concentrations were calculated from calibration done with authentic standards.

**S3 Characterization of reactor fluidics**

The liquid flow is characterized by mean velocity (*u*_m_, Eqn S1) and Reynolds number *Re* (Eqn S2.) *F* is the liquid flow rate, *A* is the cross-section area of the tube, *d* is the characteristic distance (tube diameter for free enzyme reactor: 2.1 mm; particle diameter for immobilized enzyme reactor: 0.4 mm), ρ is the density of liquid, and μ is the dynamic viscosity of the fluid at 25 °C. The density and the dynamic viscosity of the substrate solution at 25 °C were taken as 997.04 kg/m^3^ and 8.9 × 10^-4^ kg/(m∙s), respectively. The value range of *Re* is 56 - 226 and 1.6 - 6.3 for the free and the immobilized enzyme reactor, respectively. The values of *Re* indicate laminar flow under all *F* conditions used.

$u_{m}=\frac{F}{A}$ Eqn S1

$Re=\frac{u_{m} d \rho}{\mu}$ Eqn S2

The radial diffusive Peclet number *Pe_D_* (Eqn S3, also named Bodenstein number by O. Levenspiel) (Commenge et al., 2005; Levenspiel, 1999) compares the total momentum transfer to the molecular mass transfer. *D* is the coefficient of diffusion of O_2_ in the liquid. A value of *D* of 2 × 10^-9^ m^2^/s was used for calculations (Wilke and Chang, 1955). The value range for *Pe_D_* is 5 × 10^3^ - 2 × 10^4^ and 98 - 393 for the soluble enzyme reactor and the packed-bed enzyme reactor, respectively. The values indicate that the axial dispersion is controlled by convection.

${Pe}_{D}=\frac{u_{m}d}{D}$ Eqn S3

The axial dispersion for non-ideal tubular reactors under laminar flow can be expressed in terms of the Taylor-Aris axial dispersion coefficient *D_A_* (Eqn S4) (Aris, 1956; Taylor, 1953). The vessel dispersion number (whose reciprocal value is recently called longitudinal dispersive Peclet number, *Pe_D_*) can be calculated according Eqn S5. *L* is tube length. The number expresses the ratio of axial transports by dispersion and convection. Value ranges are 9 × 10 ^-3^ - 1 ×10 ^-1^ and 1.4 × 10^-3^ - 5.4 × 10^-3^ for soluble enzyme reactor and packed-bed enzyme reactor, respectively. The low values indicate prevalence of convection and low broadening of the residence time distribution (for the general case, see: Fogler, 2006; Levenspiel, 1999). Please note that for the soluble enzyme reactor, the values of *Pe_D_* and *D_A_* lie closely below the upper limit for the application of the dispersion model in a straight tube. However, here we are using a coiled tubing in which the dispersion effect is reduced due to outward radial flow. This can be expressed in terms of the Dean number *De* (Eqn S6), where R_c_ is the radius of curvature of the tube. The relevant value range of *De* is 2 - 42. It is reported that dispersion effects are reduced at *De* > 10 (Nagy et al., 2012).

$D_{A}=D+\frac{u_{m}^{2}d_{h}^{2}}{192D}$ Eqn S4

$\frac{1}{{Pe}_{L}}= \frac{D_{A}}{U_{m}L}$ Eqn S5

$De=Re \sqrt{\frac{d}{2 Rc}}$ Eqn S6

**Supporting Figures**

**
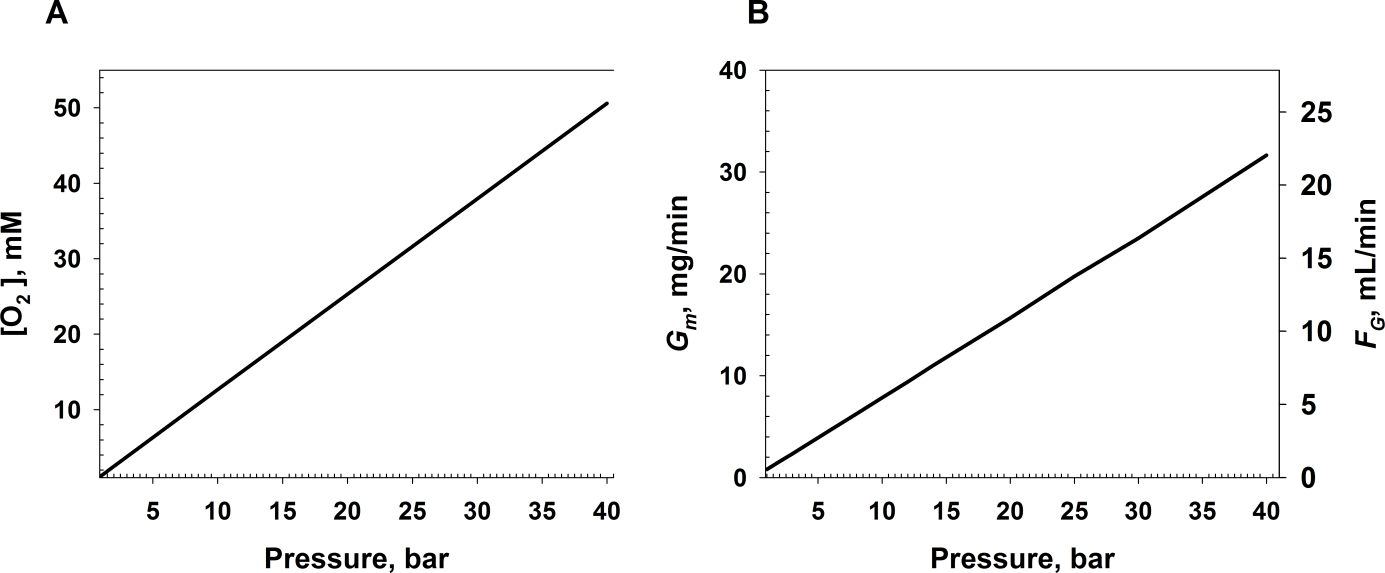
**

**Figure S1.** Levels of O_2_ saturation at different absolute operational pressures. Panel A shows the O_2_ concentration under condition of 100% O_2_ saturation at different pressures. Data were calculated using the oxygen unit calculation software from PreSens (PreSens Precision Sensing GmbH, Regensburg, DE). Panel B shows the O_2_ mass flow rate *G_m_* upon variation of pressure for a calculation baseline of *F_L_* = 20 mL/min. The value of *G_m_* needed to saturate the liquid is proportional to the liquid flow rate, *F_L_*, and the saturating [O_2_] (Panel A). The volumetric flow rate used as reference, *F_G_*, was calculated under standard conditions (Pressure_reference = 1 bar, T_ref_ = 25 ºC) using the general gas equation. The real contribution of the oxygen volumetric flow rate to the total liquid flow rate, *F*_total_, can be calculated from *F_G_*, using a compression factor proportional to Pressure/Pressure_reference. The result is that *F_G_*/*F_total_* is smaller than 2.5%.

**
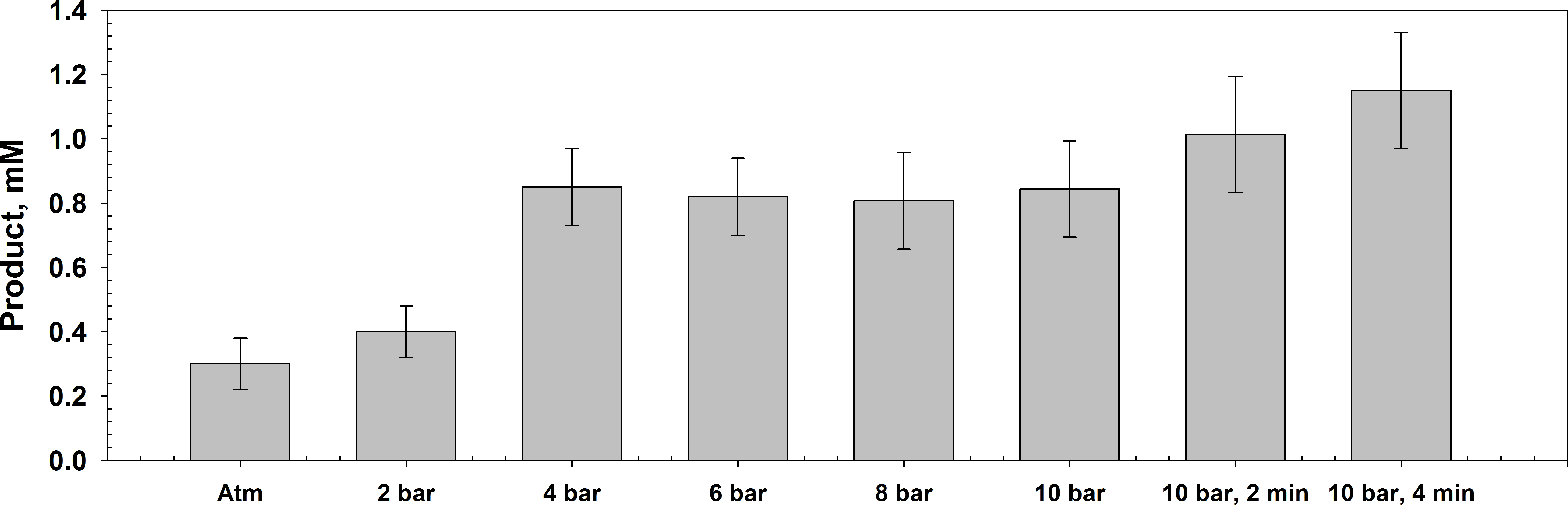
**

**Figure S2.** Product formation by free GOX in the pressurized flow reactor at different operating conditions. The initial glucose concentration was 20 mM. The *V*_ref_ was 2.5 mM/min. *V*_ref_ is the maximum reference reaction rate (see Eqn 2 of main text) at air-saturated reaction conditions and using 20 mM glucose. The τ_res_ was 1 min unless indicated in the graph.

**
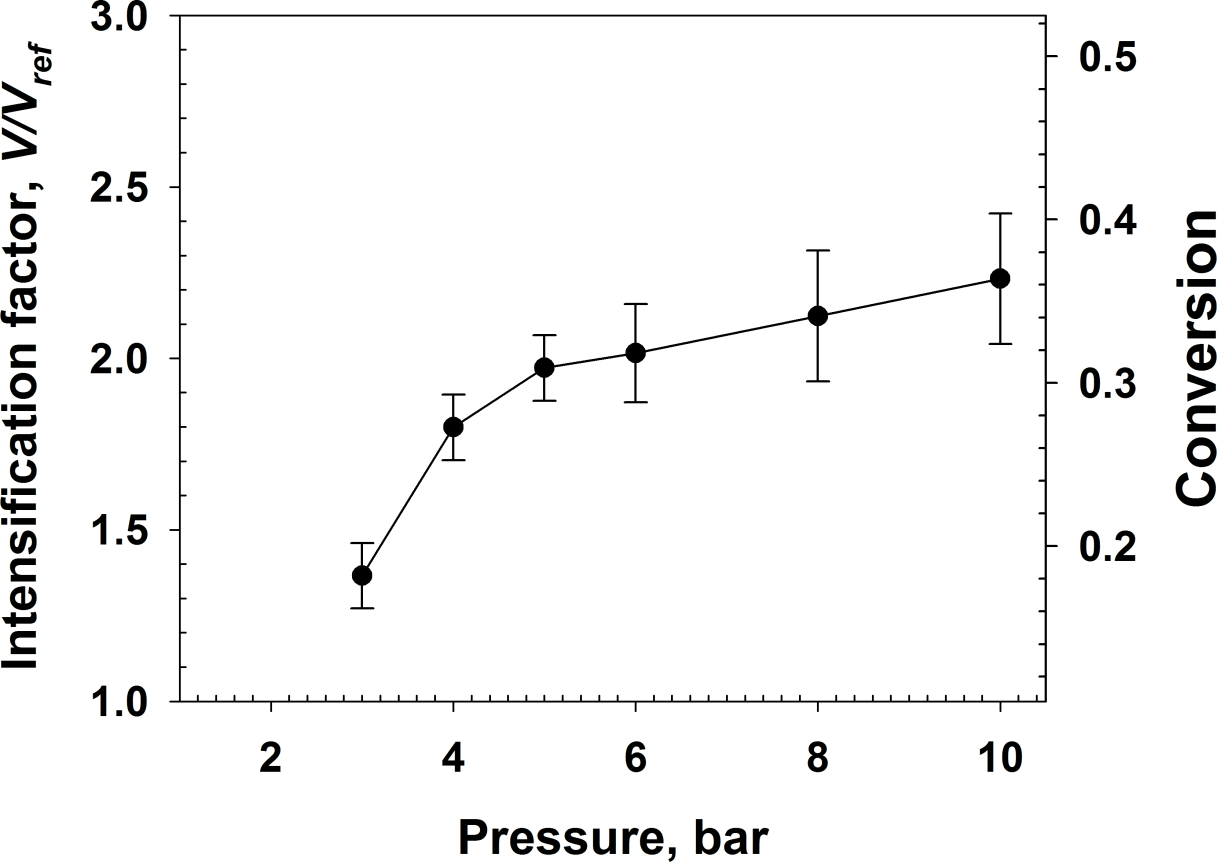
**

**Figure S3.** Conversion of glucose catalyzed by soluble GOX in the pressurized flow reactor at different pressures. Conversion is product formed/initial glucose concentration (20 mM). The intensification factor for each condition, *V/V*_ref_, is shown. *V* is the average reaction rate calculated as product formed/τ_r_. The *V*_ref_ was 2.5 mM/min. *V*_ref_ is the maximum reference reaction rate (see Eqn 2 of main text) at air-saturated conditions and using 20 mM glucose. The τ_res_ was 1 min.

**
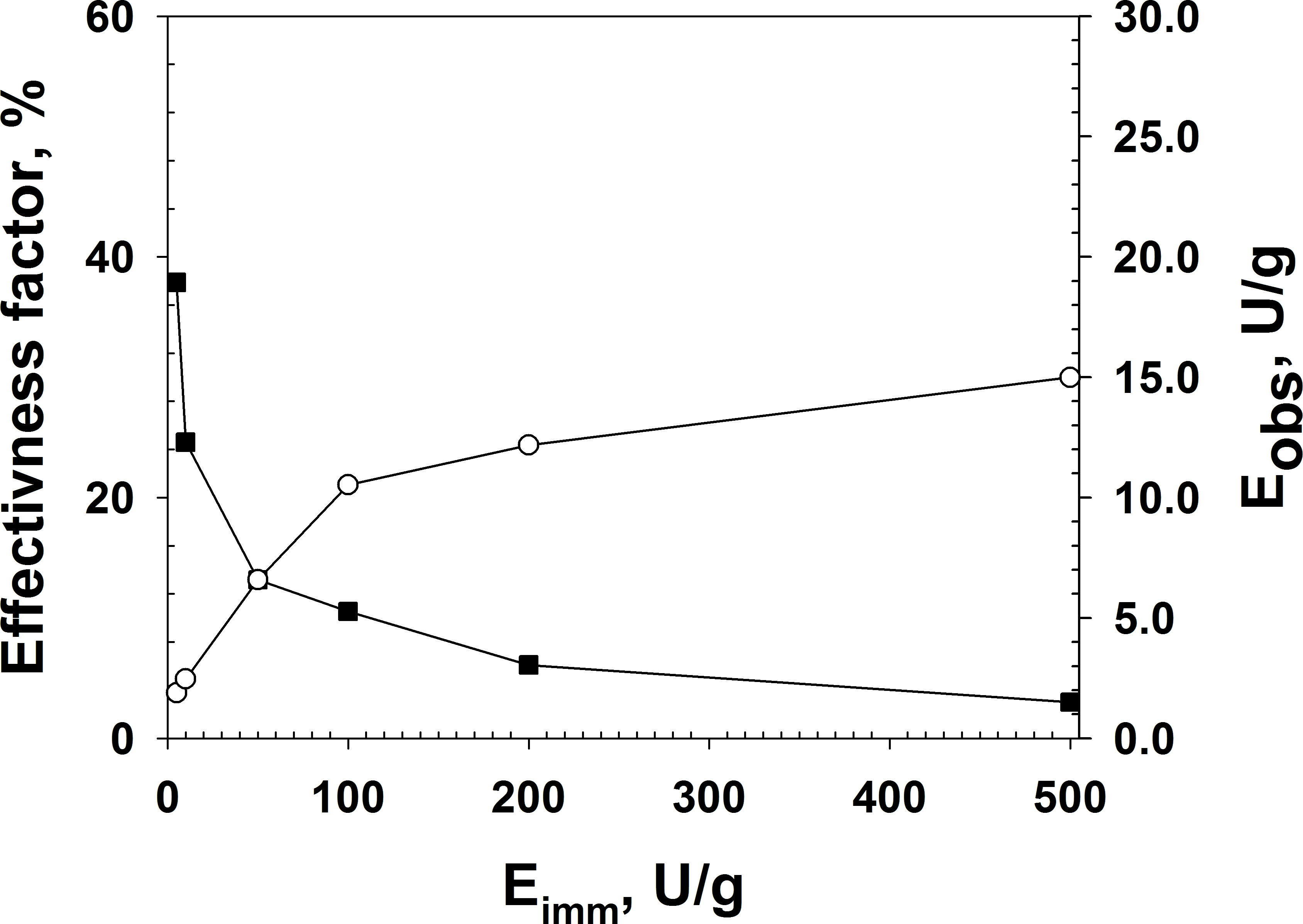
**

**Figure S4.** The observable activity E_obs_ (open circles) and the effectiveness factor (close squares) of GOX immobilizates dependent on the enzyme-to-carrier loading used. The effectiveness factor is the ratio of E_obs_ and E_imm_. E_obs_ is determined as the initial rate of O_2_ consumption in the liquid phase by the immobilized enzyme at air-saturated conditions (0.256 mM). E_imm_ is determined from the activity balance in solution before and after the immobilization.

**
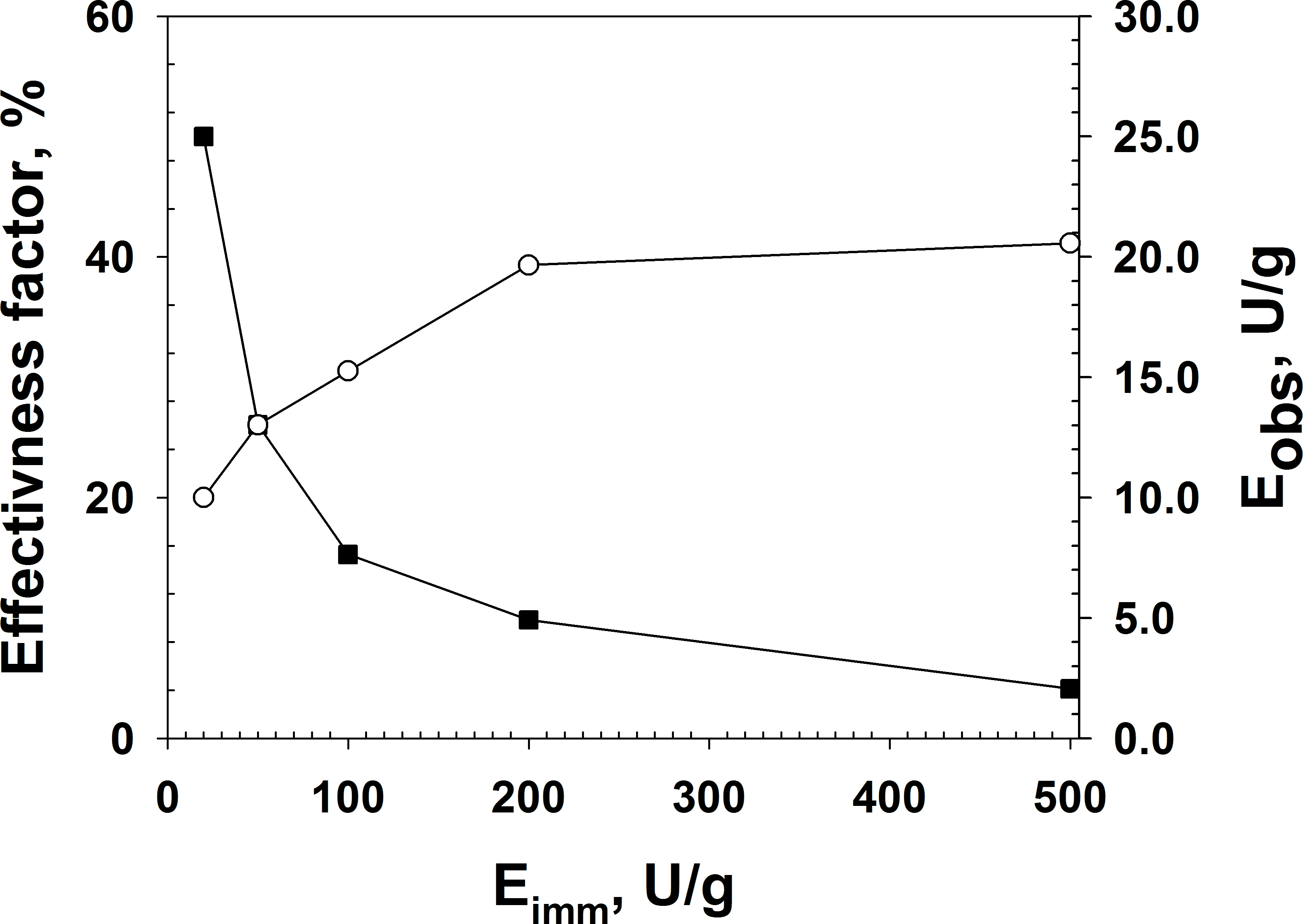
**

**Figure S5.** The observable activity E_ob_*_s_* (open circles) and the effectiveness factor (closed squares) of DAAO immobilizates dependent on the enzyme-to-carrier loading used. The effectiveness factor is the ratio of E_obs_ and E_imm_. E_obs_ is determined as the initial rate of O_2_ consumption in the liquid phase by the immobilized enzyme at air saturated conditions (0.256 mM). E_imm_ is determined from the activity balance in solution before and after the immobilization.


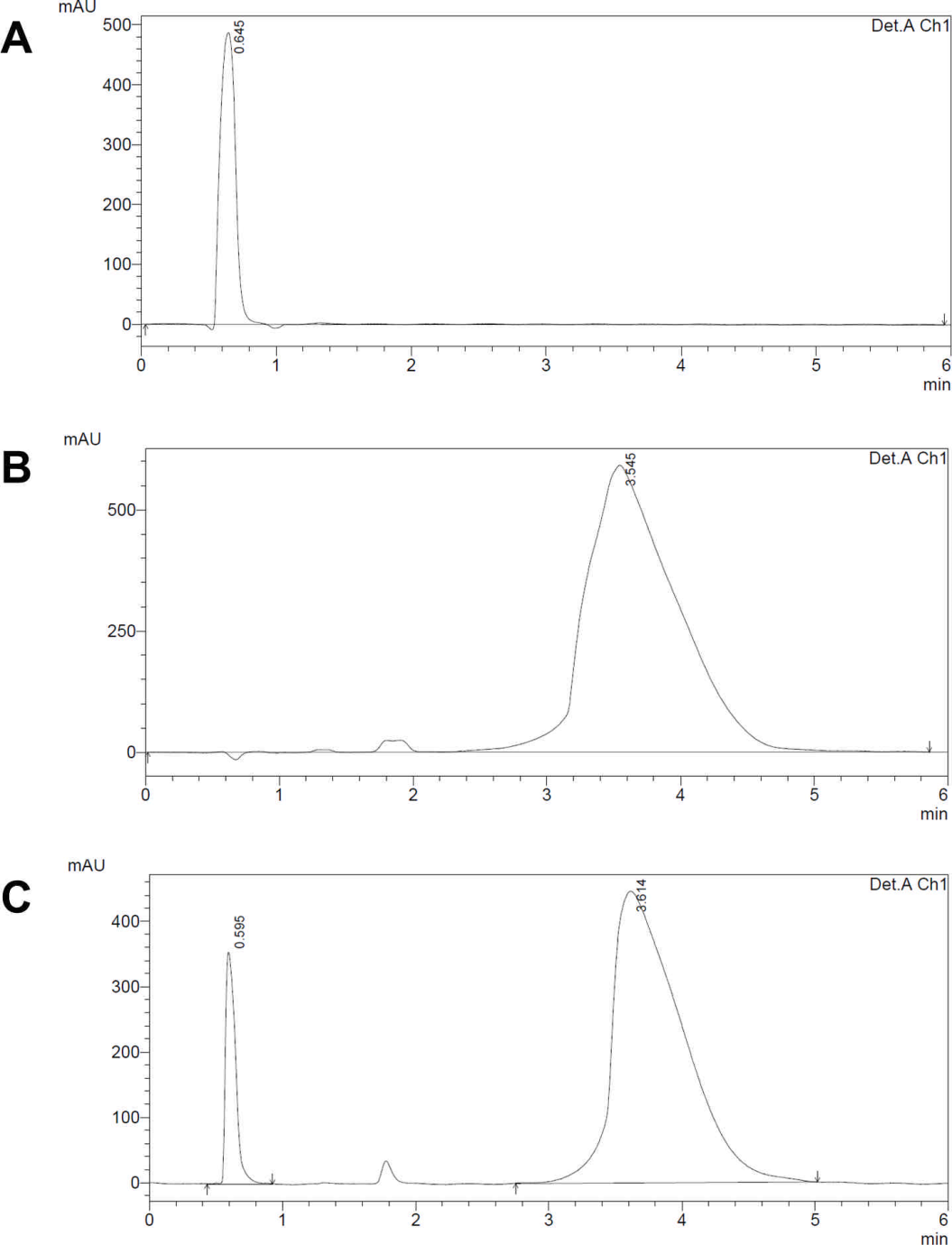


**Figure S6**. HPLC chromatogram showing the analysis of D-Met and α-keto-γ-(methylthio)butyric acid. Panel A shows the D-Met standard (retention time. 0.6 min). Panel B shows the α-keto-γ-(methylthio)butyric acid standard (retention time: 3.6 min). Panel C shows the separation of a mixture of the two standards. Note: a peak shortly before 2 min was detected from the commercial standard of α-keto-γ-(methylthio)butyric acid. Its identity was not determined.


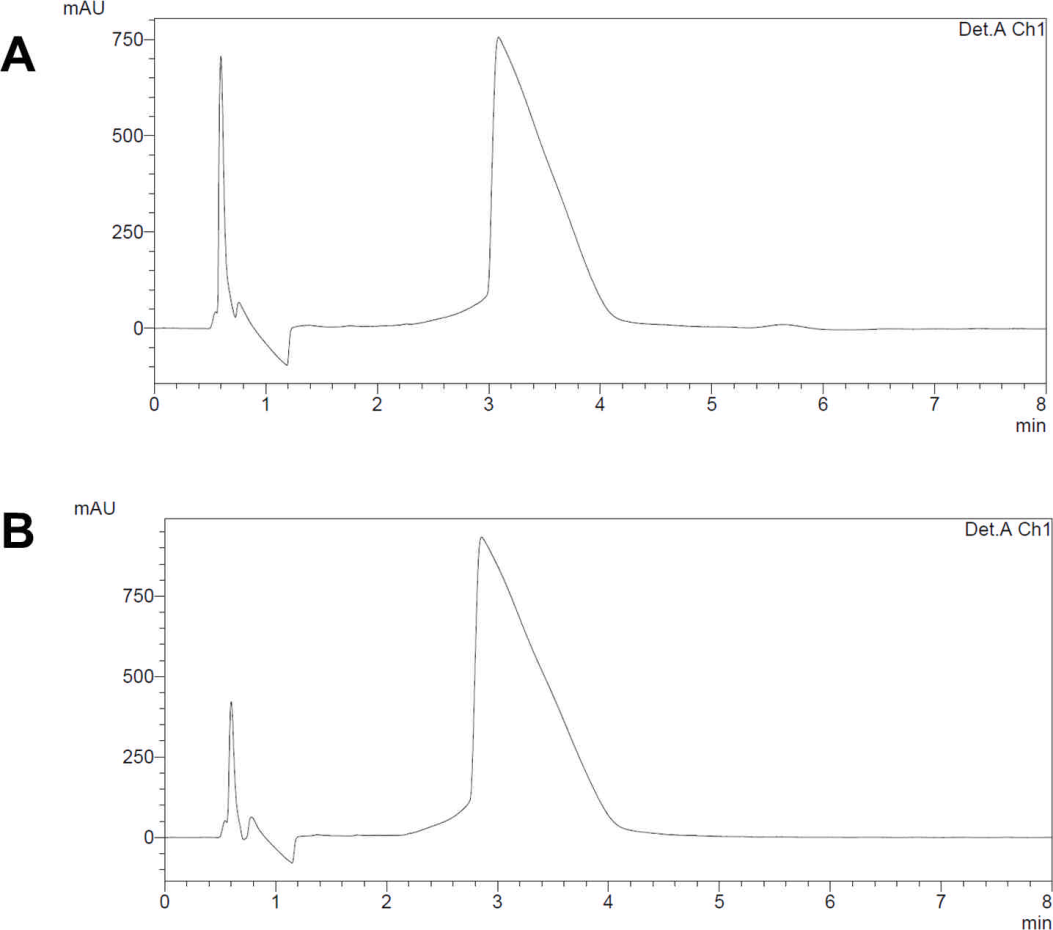


**Figure S7**. Representative HPLC chromatograms from the conversion of D-Met to α-keto-γ-(methylthio)butyric acid catalyzed by DAAO immobilizates. Panels A and B show samples at 54% and 76% conversion, respectively, based on an initial D-Met concentration of 50 mM. Note: the retention times for D-Met to α-keto-acid shifted slightly due to minor changes in the pressure/flow-rate of the mobile phase.

**
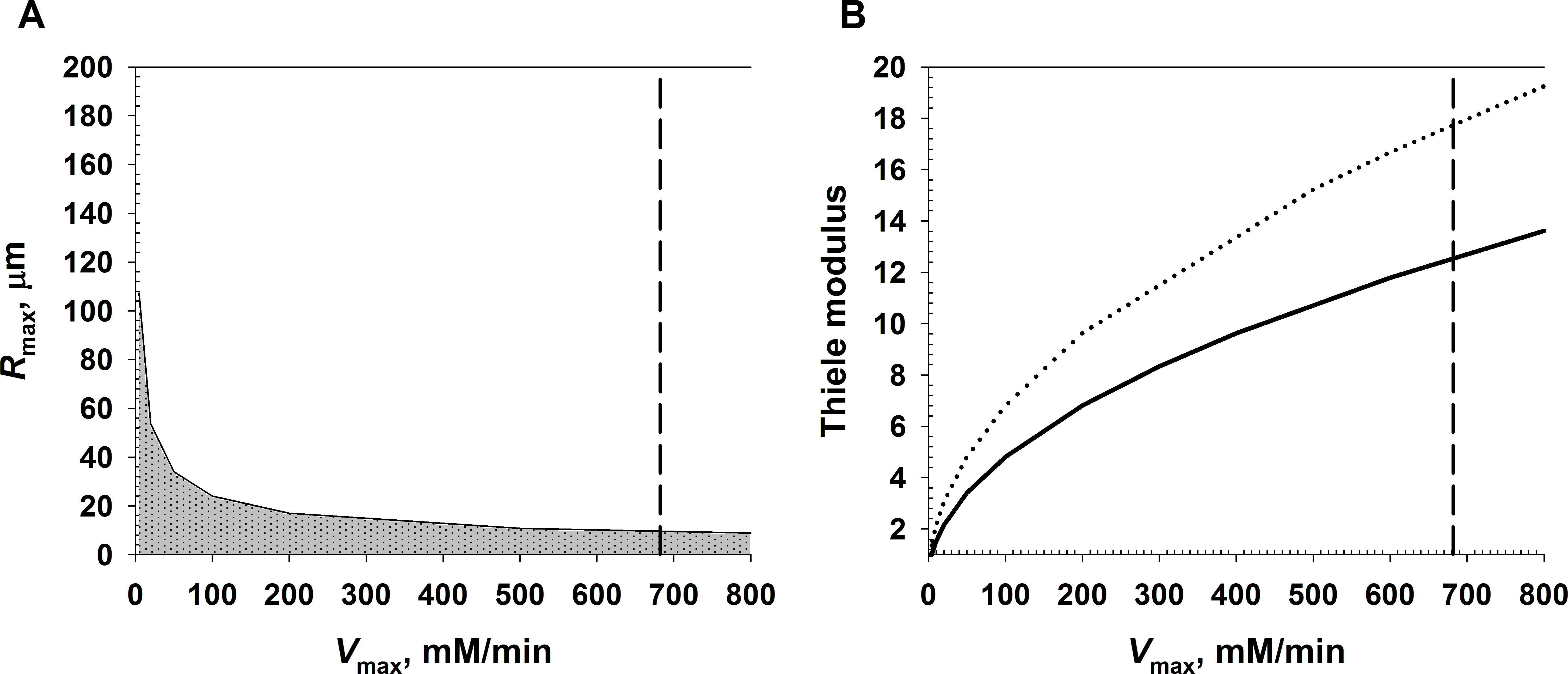
**

**Figure S8.** Chemical reaction engineering analysis of the Rel-sulfonate immobilizate of DAAO. Panel A shows the maximum radius of the particle (*R*_max_) which for a given *V*_max_ under the assumption of zero-order reaction prevents that the substrate concentration inside the particle drops to zero. The *R*_max_ dependence upon *V*_max_ was calculated ausing Eqn S7. Panel B shows the Thiele modulus, φ, for two extreme-case scenarios, first-order kinetics (dotted line) and zero-order reaction (continuous black line). φ was calculated according to Eqns S8 and S9 (Doran, 2013). The vertical dashed line indicates the experimental *V*_max_.

$R_{\max}= \sqrt{\frac{6 D_{eff}S_{0}}{V_{\max}}}$ Eqn S7

$\phi_{1st}= \frac{R}{3}\sqrt{\frac{V_{\max}}{Deff O2}}$ Eqn S8

$\phi_{zero}= \frac{R}{3 \sqrt{2}}\sqrt{\frac{V_{\max}}{Deff O2}}$ Eqn S9

*R* is the particle radius and [O_2_] is the initial O_2_ concentration (0.250 mM). *D*_eff_ _O2_ is the effective diffusion coefficient of O_2_. It was calculated using the Stokes-Einstein equation (Eqn S10). *V*_max_ is the maximum reaction rate, which according to Eqn 2 of the main text is the product of the enzyme concentration *E* and *Act*_max_. *E* (mg/mL) can be calculated from E_imm_, the specific activity of DAAO (71 U/mg), and the particle density (1.1 g/mL).

$D_{eff}=D\frac{\varepsilon}{\tau}(1-r_{molecule}/r_{pore})^{4}$ Eqn S10

In Eqn S10, *D* is the diffusion coefficient in water at 30 ºC (2 × 10^-9^ m^2^/s), ε is the particle porosity factor (0.3), and $\tau$is the tortuosity factor which is calculated using Eqn S11 (Beeckman, 1990)

$\tau^{2}= \frac{\varepsilon}{{(1-{(1-\varepsilon)}^{1/3})}^{5}}$ Eqn S11

**List of symbols**

*A*: cross-section area of the tube, m^2^

*D*: Coefficient of diffusion of O_2_, m^2^/s

*F*: volumetric flow rate, mL/min

*Re*: Reynolds number

*u*_m_: mean liquid velocity rate, m/s

*d*: characteristic length of the flow reactor, which is tube diameter for free enzyme reactor or particle diameter for packed-bed reactor, mm

*D_A_*: Taylor-Aris axial dispersion coefficient, m^2^/s

*De*: Dean number

*L*: length of the microreactor, mm

*Pe_D_*: radial diffusive Peclet number

*V*_ref_: enzymatic reaction rate obtained with an amount of enzyme *E* under reference conditions (25 ºC, air-saturated solution atmospheric pressure), μmol/(mL min)

μ: dynamic viscosity of liquid, kg m/s

ρ: density of liquid phase, kg/m^3^

**References**

Aris, R. (1956). On the dispersion of a solute in a fluid flowing through a tube. *Proceedings of the Royal Society A: Mathematical, Physical and Engineering Sciences* 235, 67–77. *doi:* 10.1098/rspa.1956.0065

Beeckman, J.W. (1990) Mathematical description of heterogeneous materials. *Chemical Engineering Science,* 45, 2603–2610. doi: 10.1016/0009-2509(90)80148-8

Boniello, C., Mayr, T., Klimant, I., Koenig, B., Riethorst, W., Nidetzky, B. (2010). Intraparticle concentration gradients for substrate and acidic product in immobilized cephalosporin C amidase and their dependencies on carrier characteristics and reaction parameters. *Biotechnology and Bioengineering,* 106, 528–540. *doi:* 10.1002/bit.22694.

Commenge, J.M., Falk, L., Corriou, J.P., Matlosz, M. (2005). Analysis of microstructured reactor characteristics for process miniaturization and intensification. *Chemical Engineering and Technology,* 28, 446–458. *doi:*[10.1002/ceat.200500017](https://doi.org/10.1002/ceat.200500017)

Doran, P.M.(2013). Heterogeneous reactions. In Doran, P.M. *Bioprocess engineering principles*. Boston, USA: Elsevier/Academic Press, pp. 721–734.

Fogler, H.S. (2006). *Elements of chemical reaction engineering*. Upper Saddle River, NJ: Prentice Hall PTR.

Levenspiel, O. (1999). *Chemical reaction engineering* 3rd ed. New York: Wiley.

Mateo, C., Abian, O., Fernandez-Lafuente, R., Guisan, J.M. (2000). Reversible enzyme immobilization via a very strong and nondistorting ionic adsorption on support-polyethylenimine composites. *Biotechnology and Bioengineering*, 68, 98–105. *doi*: 10.1002/(SICI)1097-0290(20000405)68:1<98::AID-BIT12>3.0.CO;2-T

Nagy, K.D., Shen, B., Jamison, T.F., Jensen, K.F. (2012). Mixing and dispersion in small-scale flow systems. *Organic Process Research & Development*, 16, 976–981. *doi:* 10.1021/op200349f

Taylor, G. (1953). Dispersion of soluble matter in solvent flowing slowly through a tube. *Proceedings of the Royal Society A: Mathematical, Physical and Engineering Sciences*, 219, 186–203. *doi*: 10.1098/rspa.1953.0139

Wilke, C.R., Chang, P. (1955). Correlation of diffusion coefficients in dilute solutions. *AIChE Journal* 1, 264–270. *doi*:[10.1002/aic.690010222](https://doi.org/10.1002/aic.690010222)
